# Use of Rituximab in NHL Malt Type Pregnant in I° Trimester for Two Times

**DOI:** 10.1515/med-2019-0087

**Published:** 2019-11-12

**Authors:** Antonello Sica, Paola Vitiello, Alfonso Papa, Armando Calogero, Caterina Sagnelli, Danilo Casale, Maria Mottola, Gino Svanera, Concetta Anna Dodaro, Erika Martinelli, Teresa Troiani, Fortunato Ciardiello, Beniamino Casale

**Affiliations:** 1 Department of Precision Medicine, University of Campania Luigi Vanvitelli, Naples, Italy; 2 Dermatology Unit, University of Campania Luigi Vanvitelli, Naples, Italy; 3 Pain Department, AO Dei Colli - V. Monaldi, Naples, Italy; 4 Department of Advanced Biomedical Sciences, University of Naples Federico II, Naples, Italy; 5 Department of Mental Health and Public Medicine, University of Campania Luigi Vanvitelli, Naples, Italy; 6 Department of Anesthesia, Reanimation, Intensive Care and Pain Buon Consiglio Fatebenefratelli Hospital, Naples, Italy; 7 Department of Heart Surgery and Transplantations AO Dei Colli - V. Monaldi, Naples, Italy; 8 Department of Medical Area ASLNA2 NORTH, Naples, Italy; 9 Department of Pneumology And Tisiology, AO Dei Colli - V. Monaldi, Naples, Italy

**Keywords:** Ituximab, Pregnancy and rituximab, Non-Hodgkin lymphoma

## Abstract

Administration of rituximab, one of the basic drugs for the therapy of B-cell lymphoproliferative diseases, during pregnancy has been suspected to cause developmental fetal events, particularly if given during the first trimester of pregnancy. Therefore, use in pregnancy is not permitted. Howe ver, several cases of pregnant women being treated with rituximab are reported herein; an exception is often made in cases with grave illness.

We describe an exceptional case of a woman with non-Hodgkin lymphoma of the mucosa-associated lymphoid tissue type where rituximab was given as a single agent without interruption during two consecutive pregnancies. This case can certainly supply important indications on the safety of rituximab.

## 1 Introduction

Based on human data, rituximab alleged to cause adverse fetal development: B-cell lymphocytopenia is observed in fetuses exposed to rituximab in utero [1]. Pregnant women must be informed of the risk to the fetus and those of potential childbearing age must use effective contraception during treatment with rituximab and also for 12 months after the last dose of rituximab. In pregnant women who received rituximab, the newborn infant must be observed for signs of infection [2].

Rituximab is commonly used in systemic and primitive cutaneous B-cell lymphoproliferative diseases, alone or in combination with other chemotherapy based on the type of lymphoma [3]. The most widely used chemotherapy protocols in B-cell lymphomas are rituximab, cyclophosphamide, doxorubicin hydrochloride, vincristine sulfate, and prednisone (R-CHOP).

In pregnant cynomolgus monkeys the use of rituximab during fetal organogenesis caused lymphoid B-cell depletion in the newborn. Moreover, rituximab was detected in human infants’ serum after in-utero exposure [4,5]. No data are available at present on the risk of birth defects or miscarriage. We report the first clinical case of women in whom the use of rituximab was given in two consecutive pregnancies in early months of pregnancy.

## 2 Case report

In the 01/2007, a 28-year-old Caucasian woman had her first twin pregnancy. The delivery was complicated by gestosis, with the death in utero of one of the twins and his permanence as a dead fetus in uterus for 20 days.

Two months after this event, she came for observation with eosinophilia (eosinophils 1500/μL on about WBC 10000/μL) and persistent cough. She received antibiotic therapy and on 03.26.2007 a chest X-ray examination showed an “area of faint opacification in the left median region.” On 05.07.07 a chest CT showed the following: On the left, a parenchymal consolidation area with an inhomogeneous pattern mostly with ground glass with shaded margins. Other small areas with ground glass appearance were noticeable in the apical segments of the upper lateral lobe of the middle and front-basal lobe of the right lower lobe. On 05.11.07 she underwent bronchoscopy: “…the left bronchial half system was explored, focusing on marked inflammation of the upper lobe, and in particular at the upper-dorsal branch level.”

On 06.06.07 a bone marrow biopsy was performed. The cytometric analysis of BAL, peripheral blood, and bone marrow aspirate were in agreement for CD19+, CD5-, CD22+, CD23-, CD10-, CD43+, CD11c+, lambda+ compatible with a B-cell lymphoproliferative disease. The bone marrow showed interstitial and trabecular nodular infiltration by B-cell lymphoproliferative disease. Immunohistochemical examination showed CD19 +, CD5-, CD22 +, CD23-, CD10-, CD43 +, CD11c +, lambda +, also compatible with B-cell lymphoproliferative disease. On 18.06.07 a total body positron emission tomography was performed, which “evidenced inhomogeneous accumulation of tracers with focal areas uptake corresponding to the extended parenchymal thickening with ground glass at loading the upper lobes of both lungs, as well as at the apical segment of the left lower lobe and the right middle lobe. There was evidence of faint accumulation of the tracer at the right tracheal lymph node formation but absence of additional areas of accumulation of the tracers in other body areas examined. Screening for hepatitis C virus (HCV) and hepatitis B virus (HBV) infection was performed to avoid any flare-ups during chemotherapy [6, 7, 8, 9, 10].

On 07.12.07 an esophagogastroduodenoscopy was performed: with the following results: The body and the gastric bottom are profoundly altered by extensive lesions, intense hyperemia, and multiple small ulcerations. Gastric biopsy showed moderate, active chronic gastritis with bacterial infection possibly by Helicobacter pylori and marked lymphoid reaction (small mature lymphocytes) that dissociate muscolaris mucosae, infiltrate the glandular epithelium, presenting images indicating associated lymphoid lesions. Meta-inflammatory modifications and intestinal metaplasia (10%) were observed. The lymphoid population showed marked positivity for LCA, CD19, and only poor reactivity for CD45RO for accompanying T-cells, thereby confirming the diagnosis of low-grade MALTOMA B cell. In conclusion, a diagnosis of stage IV-E (extranodal site involvement) mucosa-associated lymphoid tissue (MALT) lymphoma according to Ann Arbor staging was made.

From September 2007, she began therapy with omeprazole 20 mg, amoxicillin 1g, and either clarithromycin 500 mg twice daily for two week; dexamethasone in boluses of 40 mg / day for 4 days every 28 days; rituximab 650 mg once per week for 4 weeks. This was followed by rituximab 650 mg every 2 months given intravenously (375 mg/m^2^) for 2 years. After one week the cough disappeared. After 45 days she was evaluated: a urea breath test was negative and a CT scan examination showed a reduction of all injuries observed previously. She continued her therapeutic regime, but 10 months later amenorrhea was reported and a pregnancy test was performed with positive results. Rituximab was discontinued. However, a few days before performing the pregnancy test, we had administered 650 mg of rituximab. She decided to complete the pregnancy; prenatal screening by ultrasonography evaluated the risk for fetal malformation during the pregnancy. No fetal alterations were showed at each routine ultrasonography. The newborn baby was healthy.

After childbirth she was reevaluated with a urea breath test and CT scan that showed complete remission. The newborn experienced no adverse events; he was subjected to a strict follow-up.

She restarted rituximab 650 mg every 2 months intravenously for maintenance of the response. After 8 months, amenorrhea indicated another pregnancy. Rituximab was discontinued, but one week before performing the pregnancy test, we had administered 650 mg of rituximab. The patient again decided to complete the pregnancy, and all fetal ultrasound scans showed no alterations. The second newborn was born healthy despite being exposed to rituximab during the first trimester of pregnancy and was also subjected to a strict follow-up. Neither of the newborns ever showed any adverse events.

Human use of rituximab has been complied with all the relevant national regulations, institutional policies and in accordance the tenets of the Helsinki Declaration, and an informed consent has been obtained from patient included in this study.

## 3 Discussion

The non-Hodgkin lymphoma (NHL) MALT TYPE patient is generally associated with HP chronic infection, as in this case. This symptomless presentation has made the diagnostic path more investigative, because once we found infiltration in the BAL by MALT NHL, a gastric involvement could not be excluded. It is truly unusual how the disease has been subtle both as an expression and in symptomatology. The frequency of a first presentation in in Ann Arbor stage IV is very rare. Many cases in which rituximab was used in pregnancy have been described in the literature, but few have been used rituximab in the first trimester of pregnancy [11,12].

This is the first clinical case of a woman in whom rituximab was given in two consecutive pregnancies in first months of pregnancy. Usually, when therapy is indicated we tend to postpone treatment until after the first trimester of pregnancy because it is the most important period of organogenesis and therefore there is greater risk of malformations for the newborn. In fact, the reported cases of use rituximab during the first trimester of pregnancy are due to accidental pregnancy, similar to that which occurred in our patient.

Careful follow-up of the newborns was necessary to evaluate adverse events even after birth, and none were observed. After more than 10 years neither child showed abnormalities of the immune system, lymphocytopenia, or other anomalous predispositions to disease that could somehow involve the immune system.

## 4 Conclusion

Despite numerous reports of adverse events, and although rituximab is not a drug considered safe during pregnancy, this clinical case is certainly not to discourage all pregnant women who use rituximab and have had knowledge of unplanned pregnancy. On the other hand, it is difficult to have an in-depth knowledge of the mechanisms that generate defects in organogenesis in humans linked to the use of the drug. The behavior is not homogeneous, and the defects acquired by newborns are different. Perhaps the only defect that is described as most common is lymphocytopenia, which also characterizes adults for a long time after MAB therapy. Only reports like the present one can increase our knowledge on the subject.
